# Polarization Drift Channel Model for Coherent Fibre-Optic Systems

**DOI:** 10.1038/srep21217

**Published:** 2016-02-24

**Authors:** Cristian B. Czegledi, Magnus Karlsson, Erik Agrell, Pontus Johannisson

**Affiliations:** 1 Chalmers University of Technology, Department of Signals and Systems, SE-41296 Gothenburg, Sweden; 2 Chalmers University of Technology, Department of Microtechnology and Nanoscience, SE-41296 Gothenburg, Sweden

## Abstract

A theoretical framework is introduced to model the dynamical changes of the state of polarization during transmission in coherent fibre-optic systems. The model generalizes the one-dimensional phase noise random walk to higher dimensions, accounting for random polarization drifts, emulating a random walk on the Poincaré sphere, which has been successfully verified using experimental data. The model is described in the Jones, Stokes and real four-dimensional formalisms, and the mapping between them is derived. Such a model will be increasingly important in simulating and optimizing future systems, where polarization-multiplexed transmission and sophisticated digital signal processing will be natural parts. The proposed polarization drift model is the first of its kind as prior work either models polarization drift as a deterministic process or focuses on polarization-mode dispersion in systems where the state of polarization does not affect the receiver performance. We expect the model to be useful in a wide-range of photonics applications where stochastic polarization fluctuation is an issue.

Enabled by digital signal processing, coherent detection allows spectrally efficient communication based on quadrature amplitude modulation formats, which carry information in both the intensity and the phase of the optical field, in both polarizations. Polarization-multiplexed quadrature phase-shift keying has recently been introduced for 100 Gb/s transmission per channel and it is expected that in the near future, higher-order modulation formats will become a necessity to reach even higher data rates. However, the demultiplexing of the polarization-multiplexed channels in the receiver requires knowledge of the state of polarization (SOP), which is drifting with time. This implies that the SOP must be tracked by a dynamic equalizer^1^,^2^. As the SOP changes with time in a random fashion, it is qualitatively different from the chromatic dispersion, which can be compensated for using a static equalizer. A deterministic or static behaviour would be straightforward to resolve, but with a nondeterministic SOP drift, the equalizer must be continuously adjusted.

In order to fully understand the impact of an impairment on the performance of a transmission system, a channel model is required, which should describe the behaviour of the channel as accurately as possible. Based on the statistical information that such models reveal, insights into how to treat the impairments optimally in order to maximize performance can be obtained and used as a result. On the other hand, a channel model that does not describe the fibre accurately may hinder the achievement of optimal performance. Therefore, it is important that the channel model matches the stochastic nature of the fibre closely.

Very few results on modelling of *random* polarization drifts are present in the literature. In the context of equalizer development, several models have indeed been suggested, but typically by either using a *constant* randomly chosen SOP^3^,^4^,^5^,^6^ or by generating cyclic/quasi-cyclic *deterministic* changes^7^,^8^,^9^,^10^, which are usually nonuniform in their coverage of the possible SOPs. There is, on the other hand, a wealth of statistical models that describe differential group delay (DGD) and polarization-mode dispersion dating back to the eighties^11^. However, these results were typically developed to be applicable in systems using intensity modulation or single-polarization (differential) phase-shift keying formats, which are not affected by the SOP drift. Thus, instead of modelling the time evolution of the SOP, differential equations describing the SOP change with frequency and fibre length were typically given^12^,^13^. There are also some direct measurements of SOP changes, e.g., by Soliman *et al.*^14^. However, in their measurement, fast SOP changes were induced in a dispersion-compensating module under laboratory conditions, without considering the SOP drift of an entire fibre link. It can be concluded that stochastic SOP drift has so far not been given much attention.

This paper suggests a model for random SOP drifts in the time domain by generalizing the one-dimensional (1D) phase noise random walk to a higher dimension. The model is based on a succession of random Jones matrices, where each matrix is parameterized by three random variables, chosen from a zero-mean Gaussian distribution with a variance set by a *polarization linewidth* parameter. The latter determines the speed of the drift and depends on the system details. The polarization drift has a random walk behaviour, where each step is independent of the previous steps and equally likely in all directions. The model is given in the Jones, Stokes formalisms and in the more general real 4D formalism^15^,^16^.

As argued above, the suggested model serves a different purpose than the models of polarization-mode dispersion existing in the literature. In the latter case, the fibre is viewed either as a concatenation of a small number of segments with relatively high DGD, leading to the *hinge model*^13^,^17^,^18^, or the limiting case with segments of infinitesimal lengths, leading to a Maxwellian distribution of the DGD^19^. The most common assumption is then to model the hinges as independent random SOP rotators^13^,^18^. In the *anisotropic hinge model*, the SOP variation at the hinges is modelled as generalized waveplates parameterized by one random angle per hinge^20^. The *hybrid hinge model* is a further generalized way to model the SOP changes at the hinges^21^, but in none of these publications is the SOP time evolution discussed.

The suggested model can be used in simulations for a wide range of photonics applications, where stochastic polarization fluctuation is an issue that needs to be modelled. For example, a fibre-optic communication link can be simulated, independently of the modulated data as well as of other considered impairments, which can be useful to, e.g., characterize receivers’ performance. Moreover, it can reveal statistical knowledge of the received samples affected by polarization rotations, based on which the existing tracking algorithms can be optimally tuned or new, more powerful algorithms can be designed. High-precision transfer and remote synchronization of microwave and/or radio-frequency signals^22^ is another application that could benefit from a better understanding of how it is affected by polarization drifts, which is currently the limiting factor towards a better performance. Other applications such as fibre-optic sensors^23^ have been developed for use in a broad range of applications, fibre-optic gyroscopes^24^ and quantum key distribution^25^ are strongly affected by polarization fluctuations and may benefit from a better understanding of the transmission medium.

## Present Phase and Polarization Drift Models

The fibre propagation through a linear medium is often described by a complex 2 × 2 Jones matrix, which, neglecting the nonlinear phenomena, relates the received optical field to the input. Another approach is to use the Stokes–Mueller formalism, where the evolution of the Stokes vectors is modelled by a Mueller 3 × 3 unitary matrix. The latter has the advantage that the Stokes vectors are experimentally observable quantities and can be easily visualized as points on a 3D sphere, called the *Poincaré* sphere. A further approach to describe the SOP rotations exists in the 4D Euclidean space^15^, where the wave propagation can be described by a 4 × 4 real unitary matrix^16^. We will focus most of our discussion on the Jones formalism and connect it to the Stokes and real 4D formalisms later on.

An optical signal has two quadratures in two polarizations and can be described by a Jones vector as a function of time *t* and the propagation distance *z* as 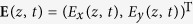, where each element combines the real and imaginary parts of the electrical field in the *x* and *y* field components and (·)^T^ denotes transposition. The transmitted signal **E**(0, *t*) into the transmission medium is obtained as 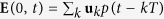 by modulating the information symbols **u**_*k*_ using a real-valued pulse shape *p*(*t*), where *T* is the symbol interval. The *k*th discrete-time received symbol 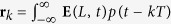 d*t* is obtained by matched filtering and sampling the received signal **E**(*L*, *t*) at distance *L*

The propagation of the optical field can be described by a 2 × 2 complex-valued Jones matrix **J**_*k*_, which relates the received optical field 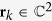, in the presence of optical amplifier noise and laser phase noise, to the input 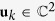 as


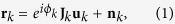


where 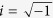, *ϕ*_*k*_ models the carrier phase noise and 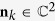 denotes the additive noise, which is represented by two independent complex circular zero-mean Gaussian random variables. Assuming that polarization-dependent losses are negligible, the channel matrix **J**_*k*_ can be modelled as a unitary matrix, which preserves the input power during propagation. In this work, we assume that the chromatic dispersion has been compensated for and polarization-mode dispersion is negligible. The transformation **J**_*k*_ can be described using the matrix function *J*(***α***_*k*_), which is expressed using the *matrix exponential* [ref. 26, p. 165] parameterized by three variables 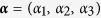 according to^27^,^28^


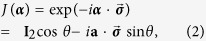


where 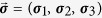 is a tensor of the Pauli spin matrices^28^


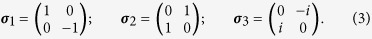


This notation of ***σ***_*i*_ complies with the definition of the Stokes vector, and it is different from the notation introduced by Frigo^27^. The operation 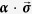 should be interpreted as a linear combination of the three matrices ***σ***_1_, ***σ***_2_, ***σ***_3_, and **I**_2_ is the 2 × 2 identity matrix. In general, a 2 × 2 complex unitary matrix has four degrees of freedom (DOFs), but in this case we explicitly factored out the phase noise. Including the identity matrix in 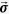 would make it possible to account for all four DOFs. The vector ***α*** can be expressed as a product ***α*** = *θ***a**, of its length 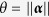 in the interval 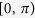 and the unit vector 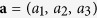, which represents its direction on the unit sphere, where ‖·‖ denotes the Euclidean norm. Since **J**_*k*_ is unitary, the inverse can be found by the conjugate transpose operation or by negating ***α***_*k*_, since 
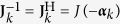
. The same principle holds for the phase noise 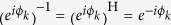.

Modern coherent systems require a local oscillator at the receiver in order to get access to both phase and amplitude of the electrical field. The local oscillator serves as a reference but it is not synchronized with the transmitter laser, resulting in phase noise, which creates the need for carrier synchronization. The phase noise is modelled by the angle *ϕ*, while *α*_1_, *α*_2_ and *α*_3_ model random fluctuations of the SOP caused by fibre birefringence and coupling. Both phase noise and SOP drift are dynamical processes that change randomly over time. The SOP drift time can vary from microseconds up to seconds, depending on the link type. It is usually much longer than the phase drift time, which is in the microsecond range for modern coherent systems^29^.

The update rule of the phase noise follows a Wiener process^30^,^31^,^32^


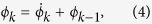


where 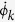 is the *innovation* of the phase noise. An alternative form of equation (4) is


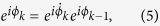


which we will generalize later to account for the SOP drift. The innovation 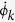 is a random variable drawn independently at each time instance *k* from a zero-mean Gaussian distribution


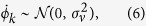


where 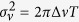 and Δ*v* is the sum of the linewidths of the transmitter and local oscillator lasers.

The accumulated phase noise at time *k* is the summation of *k* Gaussian random variables 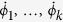 and the initial phase *ϕ*_0_, which becomes a Gaussian-distributed random variable with mean *ϕ*_0_ and variance 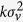. The function 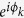 is periodic with period 2*π*, which means that the phase angle *ϕ*_*k*_ can be limited to the interval 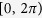 by applying the modulo 2*π* operation. In this case, the probability density function (pdf) of *ϕ*_*k*_ becomes a wrapped (around the unit circle) Gaussian distribution. It can be straightforwardly verified that the phase drift model has the following properties:

1. The innovation 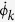 is *independent* of 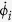 for 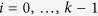.
2. The innovation is *symmetric*, in the sense that the probabilities for phase changes *ϕ* and −*ϕ* are equally likely.
3. The most likely next phase state is the current state.
4. The outcome of two consecutive steps 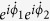 can be emulated by a single step 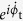 by doubling the variance of 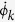.
5. As *k* increases, the distribution of 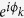 will approach the *uniform distribution* on the unit circle. The convergence rate towards this distribution increases with the Δ*vT* product.

**Figure 1:**
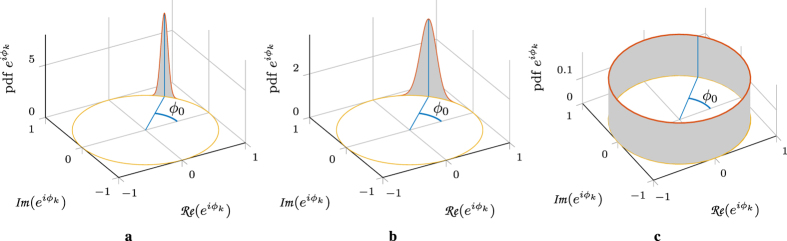
Phase noise pdf evolution. The pdf of 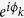 for 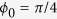 and 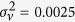 is shown. In (**a**), *k* = 1 corresponds to a single innovation and illustrates the second and third properties, i.e., the pdf is symmetric around the current state (the vertical line) and the peak of the pdf is at the current state. In (**b**), *k* = 5 and the pdf spreads over the circle. In (**c**), *k* = 8000 and the pdf approaches the uniform pdf, which supports the last property.

The time evolution of the pdf of a fixed point corrupted by phase noise is exemplified in Fig. 1.

The initial phase difference between the two free running lasers has equal probability for every value, therefore it is common to model *ϕ*_0_ as a random variable uniformly distributed in the interval 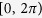, i.e., it has *equal probability* for every possible state.

The autocorrelation function (ACF) quantifies the level of correlation between two samples of a random process taken at different time instances by taking the expected value 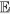
[·] of the product of the samples. The ACF of the phase noise with *lT* time separation in response to a constant input **u** is^33^


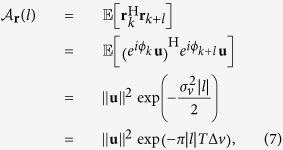


where the operations |·| denotes the absolute value. Here we neglected the SOP drift given by **J**_*k*_, in order to isolate the effects of the phase noise. We will compute the ACF of the SOP drift below.

In the literature, polarization rotations are generally modelled by the Jones matrix **J**_*k*_ in equation (1) or subsets of it, obtained by considering only one or two of the three DOFs *α*_1_, *α*_2_, *α*_3_. Contrary to the phase noise, which is a random walk with respect to time, the matrix **J**_*k*_ is in previous literature usually kept *constant*^3^,^4^,^5^,^6^,^34^, or it follows a *deterministic* cyclic/quasi-cyclic rotation pattern. The latter is obtained by modelling the parameters of **J**_*k*_ as frequency components *ωkT*. For example, *α*_3_ = *ωkT*^8^,^9^,^35^ or *α*_2_, *α*_3_ varied at different frequencies^2^,^7^,^10^.

## Proposed Polarization Drift Model

We propose to model the polarization drift as a sequence of *random* matrices, which exploit all *three* DOFs of **J**_*k*_. The model simulates a random walk on the Poincaré sphere and we describe it in the Jones, Stokes and real 4D formalisms. In general, 4D unitary matrices have *six* DOFs, spanning a richer space than the Jones (four DOFs) or Mueller (three DOFs) matrices can. Out of the six DOFs, only four are physically realizable for propagating photons, and the remaining two are impossible to describe in the Jones or Stokes space^16^. The Jones formalism can describe any physically possible phenomenon in the optical fibre making it sufficient for wave propagation. The 4D representation is preferred in some digital communication scenarios since the performance of a constellation corrupted by additive noise can be directly quantified in this space in contrast to the Stokes formalism. Even though the extra two DOFs do not model lightwave propagation, they can be used to account for transmitter and/or receiver imperfections, which cannot be done using Jones or Mueller matrices. However, the extra two DOFs are out of the scope of this paper and we will focus on the remaining four.

### Jones Space Description

Similarly to the phase noise update equation (5), we model the time evolution of **J**_*k*_ as


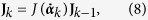


where 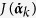 is a random *innovation* matrix defined as equation (2). This mathematical formulation is not new to the polarization community, as it is commonly used to describe the polarization evolution in space (each Jones matrix represents a waveplate). However, here it models the temporal evolution of the SOP, where each innovation matrix corresponds to a time increment. The parameters of the innovation 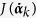 are random and drawn independently from a zero-mean Gaussian distribution at each time instance *k*


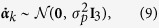


where 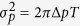, and we refer to Δ*p* as the *polarization linewidth*, which quantifies the speed of the SOP drift, analogous to the linewidth describing the phase noise, cf. equation (6). Drawing ***α*** from a zero-mean Gaussian distribution results in special cases of *θ* and **a**, where the former becomes a Maxwell–Boltzmann distributed random variable, and the vector **a** is *uniformly* distributed over the 3D unit sphere, implying that the marginal distribution of each *a*_*i*_ is uniform in [−1, 1]^36^.

It is important to note that, contrary to phase noise, the equivalent vector ***α***_*k*_ parameterizing **J**_*k*_ in equation (8) does not follow a Wiener process, i.e., 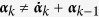, because in general 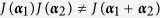. Equality occurs for (anti-)parallel ***α***_1_ and ***α***_2_, and holds approximately when 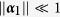 and 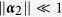. In a prestudy for this work^37^, we incorrectly used a Wiener process model for the polarization drift. We will return to that model later and discuss its shortcomings.

### Stokes Space Description

The evolution of the SOP is often analysed in the Stokes space, where the Jones vectors are expressed as real 3D Stokes vectors and can be easily visualized on the Poincaré sphere. The transmitted Jones vector **u**_*k*_ can be expressed as a Stokes vector^38^, eq. (2.5.26)


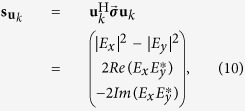


where the *i*th component of 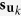 is given by 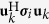 and 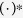 denotes conjugation. The equivalent Stokes propagation model of equation (1) can be written


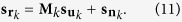


It is important to note that only 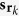 and 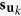 can be obtained by applying equation (10) to **r**_*k*_ and **u**_*k*_, respectively. The noise component 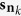 consists of three terms


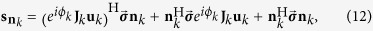


where the first two represent the signal–noise interaction and the last one the noise--noise interaction. It should be noted that there is no time averaging in equation (10) and it represents an instantaneous mapping to the Stokes space. Thus, the noise terms are polarized and rapidly varying.

The matrix **M**_*k*_ is a 3 × 3 Mueller matrix, corresponding to the Jones matrix **J**_*k*_, and the polarization transformation introduced by it can be seen as a *rotation* of the Poincaré sphere. It has three parameters, the same as **J**_*k*_, and can be written as **M**_*k*_ = *M*(***α***_*k*_), where the function 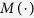 can be expressed using the matrix exponential^28^


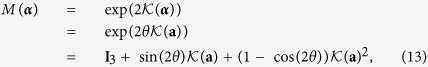


where 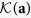 denotes


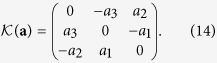


The inverse can be obtained by 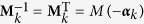. The transformation in equation (13) can be viewed in the axis-angle rotation description as a rotation around the unit vector **a** by an angle 2*θ*.

Note that any operation that applies a phase rotation on both polarizations with the same amount, such as phase noise or frequency offsets, *cannot* be modelled in the Stokes space. This can be seen in equation (11), where the phase noise does not alter the transmitted Stokes vector directly, but only contributes to the additive noise in equation (12), which would not exist in the absence of **n**_*k*_.

Analogously to equation (8), the time evolution of **M**_*k*_ is


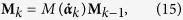


where 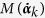 is the innovation matrix parameterized by the random vector 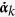 defined in equation (9).

**Figure 2:**
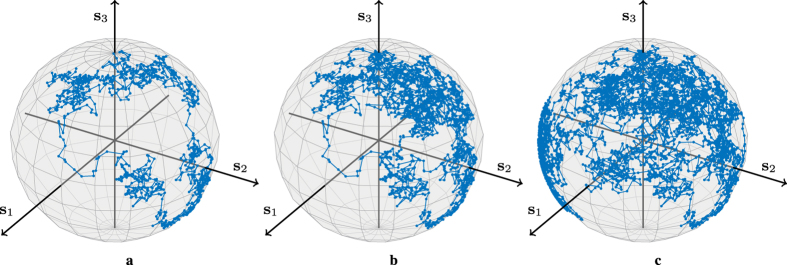
Random walk. The evolution of a random SOP drift obtained by equation (11), without additive noise, for a fixed input **s**_**u**_ = (0, 0, 1)^T^ and 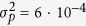 is shown. The trajectories for (**a**) 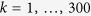, (**b**) 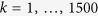 and (**c**) 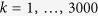 are plotted.

Figure 2 shows an example of an SOP drift as it evolves with time. The line represents the evolution of the vector **M**_*k*_(0, 0, 1)^T^ for 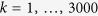.

### 4D Real Space Description

Another approach to express the time evolution of the phase and polarization drifts is to use the more uncommon 4D real formalism^15^,^16^,^39^,^40^,^41^. In this case, the transmitted/received symbol and the noise term in equation (1) can all be represented as a real four-component vector 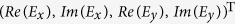. The transformations induced by the channel are modelled by 4 × 4 real unitary matrices. The 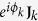 transformation in equation (1) can be combined into


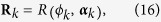


and the function *R*(·) can be described using the matrix exponential of a linear combination of four basis matrices^16^


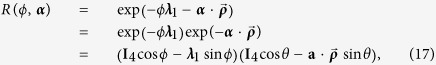


where 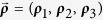 and ***λ***_1_ are four constant basis matrices^16^


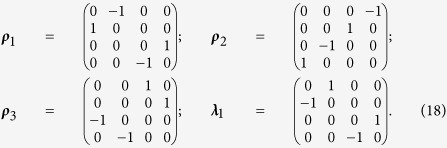


The inverse can be obtained as 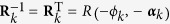. The update of **R**_*k*_ in equation (16) can be expressed analogously to equations (8) and (15) as


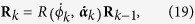


where the phase innovation 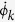 and the random vector 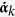 are defined by equations (6) and (9), respectively.

## Polarization Drift Model Properties

Due to the similarities between the phase noise and the SOP drift, we will use the properties of the phase noise previously presented as guidelines to validate the proposed channel model. These properties can be reformulated as follows:

1. The innovation 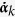 is *independent* of 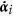 for 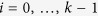.
2. The innovation is *isotropic*, in the sense that all possible orientations of the changes of the Stokes vector corresponding to a movement given by one innovation are equally likely.
3. The most likely next SOP is the current state.
4. The outcome of two consecutive steps 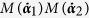 can be emulated by a single step 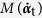 by doubling the variance 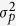 of 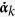.
5. As *k* increases, the pdf of the product 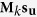, for a constant **s**_**u**_, will approach the *uniform distribution* on the Poincaré sphere. The convergence rate towards this distribution increases with the Δ*pT* product.

In the following, we will investigate these properties and discuss their validity.

Independent Innovations: This can be easily concluded from the updating rule in equation (8), (15) or (19), where the parameters of the innovation do not depend on neither the previous innovations nor the state.

### 

Isotropic Innovation: We will use the following theorem to show that the innovation 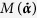 is isotropic.

**Theorem 1.** *Let a random unit vector* 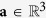 *be uniformly distributed over the 3D sphere, γ be a random angle with an arbitrary pdf and* 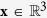 *an arbitrary unit vector. The pdf of the vector* 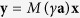
*, where* 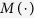 *is defined in* equation (13)*, is invariant to rotations around* **x***, i.e.,* 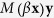 *has the same pdf regardless of β.*

In other words, the theorem states that the pdf of the random vector 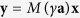 does not change if it is rotated by any angle around **x**. This can be true only if **y** is isotropic (centred and equally likely in all directions) around **x**. The technical details of the proof are presented in Supplementary Section I.

Note that Theorem 1 holds for our proposed Stokes innovation matrix 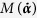 since 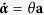 and **a** is uniformly distributed over the 3D sphere, hence the vector 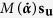 is isotropic around **s**_**u**_. The evolution of **M**_*k*_**s**_**u**_ can be seen as an *isotropic random walk* on the Poincaré sphere starting at **M**_0_**s**_**u**_ and taking random steps, of size proportional to *σ*_*p*_, equally likely in all directions. Curiously, the isotropic property is given *only* by the fact that the rotation axis **a** is uniformly distributed over the sphere, while the angle *θ* may have *any* pdf. In fact, the pdf of the angle *θ* determines the shape of the pdf of 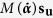, which we will discuss later. In contrast, our preliminary SOP drift model^37^ does not fulfil the isotropicity condition. The update method of the channel matrix was done by modelling ***α***_*k*_ as Wiener processes, which does not fulfil Theorem 1.

### 

Pdf of a Random Step: Unfortunately, we were not able to find a closed form expression of the pdf of the point 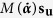 for a fixed **s**_**u**_ and a random 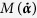. Using approximations, valid for 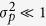, which is the case for most practical scenarios, the pdf of 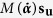 can be approximated by a bivariate Gaussian pdf centred at **s**_**u**_ of variance 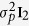 on the plane normal to **s**_**u**_. The peak of the pdf is located at **s**_**u**_ and we can conclude that the next most likely SOP is the current one. The details of the derivations are provided in Supplementary Section II. In the same section, under the same assumption of small 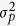, we show that the outcome of two consecutive random innovations can be achieved by a single random innovation if the variance of the random variables 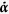 is doubled, which fulfils property 4.

### 

Uniformity: The point **M**_*k*_**s**_**u**_ performs an isotropic random walk on the Poincaré sphere, therefore as the number of steps *k* increases, the coverage of the sphere will approach uniformity, meaning that all SOPs will be equally likely. This is intuitive because taking random steps in all directions with no preferred orientation will lead to a uniform coverage. This property was observed by Zhang *et al.*^42^, where measurements of a submarine cable resulted in uniform converge of the Poincaré sphere.

**Figure 3:**
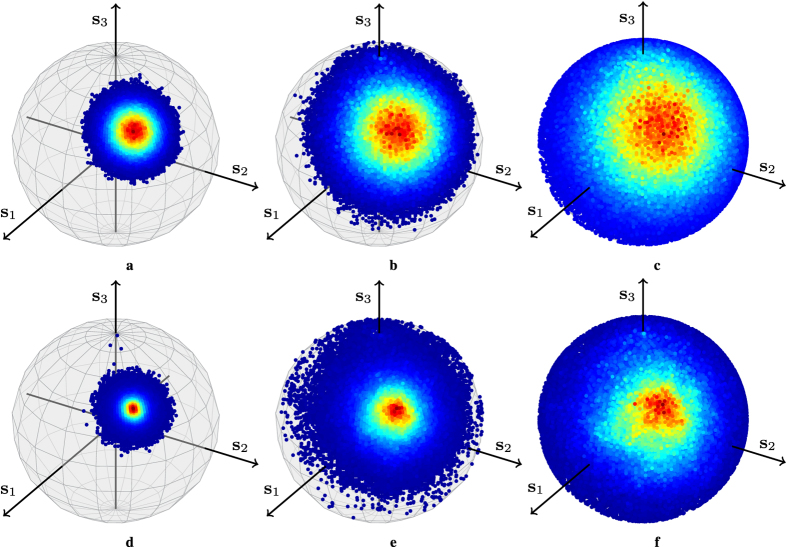
SOP drift pdf evolution. The histograms of M _*k*_s_u_ for different steps *k* and a fixed 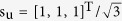 obtained from the model (top row) and from measurements (bottom row) are shown. The highest density is represented by dark red and the lowest by dark blue, the outer part of the density. The parameters of the simulated drift in equation (9) are *T* = 2.2 h (set by the measurements) and Δ*p* = 60 nHz (obtained by fitting the dash-dotted ACF line in Fig. 4). In (**a**,**d**), *k* = 2 innovation steps are plotted, whereas *k* = 8 in (**b**,**e**) and *k* = 16 in (**c**,**f**). Gaussian-like isotropic distributions can be noted in all cases, simulations and measurements, leading to a good (visual) agreement. The spread over the sphere increases with *k* and the pdf will become uniform if we let *k* grow large enough. Unfortunately, our measured data do not cover a long enough time period such that uniformity is achieved.

We compare the model with experimental results in Fig. 3, where the evolution of the pdf of a Stokes vector affected by polarization drift after different numbers of innovations starting from a fixed point is shown. The experimental data was obtained by measuring the Jones matrices of a 127 km long buried fibre from 1505 nm to 1565 nm in steps of 0.1 nm for 36 days at every ~2.2 h. The technicalities of the measurement setup and postprocessing have been published elsewhere^43^. The histograms corresponding to the measurements were captured from all the Stokes vectors obtained by applying equation (10) to the product of the matrix 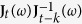, i.e., the measured matrix corresponding to *k* innovation steps, with a constant Jones vector, where **J**_*t*_(*ω*) denotes the measured Jones matrix at time *t* and wavelength *ω*.

#### Initial State

The initial channel matrix **M**_0_ should be chosen such that all the SOPs on the Poincaré sphere are *uniformly* distributed, i.e., equally likely, after the initial step **M**_0_**s**_**u**_, for any **s**_**u**_. Analogously, the initial phase *ϕ*_0_ in equation (4) should be chosen from a uniform distribution in the interval 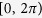. In order to generate such a matrix **M**_0_, the axis **a** must be uniformly distributed over the unit sphere and the distribution of the angle 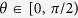 must be 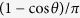
44. The generation of the angle *θ* is not straightforward, and therefore we present an alternative to generate the axis and the angle simultaneously^41^. The vector ***α***_0_ = *θ***a** is formed from the unit vector 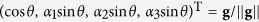, where 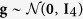, which will satisfy the conditions for both axis **a** and angle *θ*. This approach of generating ***α***_0_ of the initial channel matrix can be used regardless of the considered method to characterize the polarization evolution, i.e., Jones, Stokes or real 4D formalism.

#### The SOP Autocorrelation Functions

The ACF is commonly used to quantify how quickly, on average, the channel changes in time, frequency, etc.^45^,^46^,^47^. The ACF of the SOP drift separated by a time difference of *lT* in response to a constant input **u** can be expressed as


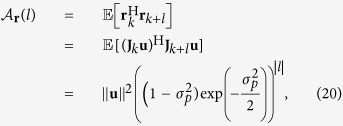


where **r**_*k*_ = **J**_*k*_**u** is the received symbol. The details of this derivation can be found in Supplementary Section III. Using the approximation 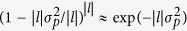, which is valid for 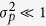, equation (20) can be approximated as


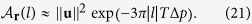


The ACF expressions in equations (20) and (21) hold for the Jones and real 4D space descriptions. Using an analogous derivation as for equation (20), it can be shown that the ACF of the SOP drift in the Stokes space is


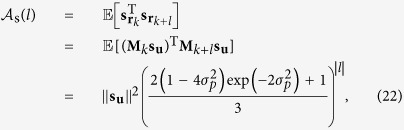


where 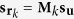 is the Stokes received symbol of a constant input **s**_**u**_ affected by polarization drift. Using the following approximations: 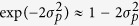, 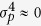 and 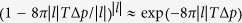 for 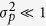, it can be approximated as


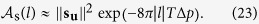


Both ACFs of the polarization drift depend only on the time separation *l* but not on the absolute time *k*. Comparing equation (21) with (23), it is interesting to note that even if they describe the same physical phenomenon, on average, a small movement in the Jones/real 4D space will result in a movement that is 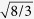 larger in the Stokes space. This result was also previously observed in similar setups analysing polarization-mode dispersion, where the autocorrelation, with respect to frequency, was derived^43^,^45^. The ACF of the phase noise equation (7) has the slowest decay rate, being a factor of three slower than equation (21), and a factor of eight slower than equation (23).

**Figure 4:**
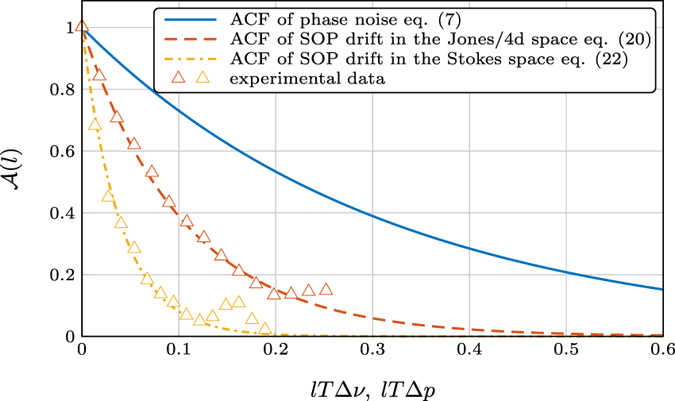
ACF comparison. The normalized ACF of the phase noise and SOP drift is plotted versus normalized time. Solid/dashed lines refer to the analytic expressions, whereas the triangles are extracted from measurements. We observe excellent agreement between the experiment and theory, except in the tails of the experimental ACF. This inconsistency can be caused by the lack of accuracy in that region.

Figure 4 shows the ACFs of the phase noise and the SOP drift, and the later is compared with experimental results. Here the analytic equations (20) and (22) match the experiment for Δ*p* = 80 nHz and Δ*p* = 60 nHz, respectively, and *T* = 2.2 h. We attribute the discrepancy between the two values to the nonunitary of the measured Jones matrices, which, through the nonlinear operation in equation (10), cause the inconsistency.

#### Linewidth Parameters

The choice of Δ*v* and Δ*p* is important when a system is simulated considering phase noise and/or SOP drifts. Both parameters reflect physical properties of the system. The quality of the (transmitter and receiver) lasers can be quantified by the Δ*v* parameter, which represents the sum of the linewidths of the deployed lasers. Distributed feedback lasers are commonly employed in transmission systems due to their cost efficiency. The linewidth of such lasers varies from 100 kHz to 10 MHz^30^. The polarization linewidth parameter depends on the installation details. Measurements of polarization fluctuations have been reported varying from weeks (this work) to seconds^48^, milliseconds^49^,^50^ or microseconds under mechanical perturbations^51^. The polarization linewidth could be quantified through measurements of the ACF, either in the Jones or Stokes space, as in Fig. 4.

## Summary

This section is intended to summarize the previous sections and provide an easy implementable form of the proposed model without requiring knowledge about the details of the derivations.

First, the parameters of the channel must be selected:

- The symbol time *T*.
- The laser linewidth Δ*v*, where the contributions of the transmitter and receiver lasers are taken into account.
- The polarization linewidth Δ*p*.

The model is then implemented as follows:

- 0. Set the initial state of the channel:

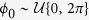
.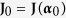 using equation (2), where ***α***_0_ = *θ***a**; *θ* and **a** = (*a*_1_, *a*_2_, *a*_3_) are identified from the unit vector 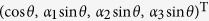 = **g**/||**g**||, where 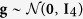.

- 1. For *k* ≥ 1, update the channel state:

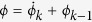
, where 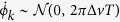.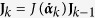, where 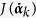 is given by equation (2) and 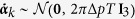.

- 2. Apply the model for every transmitted symbol **u**_*k*_, which results in the received symbol **r**_*k*_:

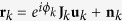
, where **n**_*k*_ denotes the additive noise represented by two independent complex circular zero-mean Gaussian random variables.

Repeat the last two steps for every symbol **u**_*k*_.

Alternatively, the Jones formalism used above can be replaced by the Stokes formalism using equation (13) instead of equation (2) and neglecting the phase noise 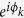; or 4D formalism by combining 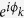 and **J**_*k*_ into **R**_*k*_ given by equation (17). In the former case, **u**_*k*_ and **r**_*k*_ are 3D Stokes vectors defined as equation (10), whereas in the latter case, **u**_*k*_, **r**_*k*_ and **n**_*k*_ are 4D real vectors.

In the summary above, we have included phase and additive noise for completeness. Without loss of generality, the model can be applied with/without phase and additive noise; also other impairments can be considered.

## Conclusions

We have proposed a channel model to emulate random polarization fluctuations based on a sequence of random matrices. The model is presented in the three formalisms, Jones, Stokes and real 4D, and it is parameterized by a single variable, called the polarization linewidth.

The model has an isotropic behaviour, which has been successfully verified using experimental data, where every step on the Poincaré sphere is equally likely in all directions emulating an isotropic random walk and can be easily coupled with any other impairments to form a complete channel model.

Compared to the existing literature, the fundamental advantages of the proposed model are randomness and statistical uniformity. Such a model is relevant for a wide range of fibre-optical applications where stochastic polarization fluctuations are an issue. It can potentially lead to improved signal processing that accounts optimally for this impairment and more realistic simulations can be carried out in order to accurately quantify system performance.

## Additional Information

**How to cite this article**: Czegledi, C. B. *et al.* Polarization Drift Channel Model for Coherent Fibre-Optic Systems. *Sci. Rep.* **6**, 21217; doi: 10.1038/srep21217 (2016).

## Supplementary Material

Supplementary Information
