# Supplementary material for: Polarization Drift Channel Model for Coherent Fibre-Optic Systems
Source: Sci Rep. 2016 Feb 24;6:21217. doi: 10.1038/srep21217 (PMC4764918; doi:10.1038/srep21217)
Supplement: Supplementary Information [file srep21217-s1.pdf]

Polarization Drift Channel Model for Coherent Fibre-Optic Systems

Supplementary Information

Cristian B. Czegledi, Magnus Karlsson, Erik Agrell
and Pontus Johannisson

I Isotropicity

We will first prove a lemma, which will be used to prove Theorem 1 in the main paper.

Lemma 1. *For all unit vectors* $\mathbf{a}_1, \mathbf{a}_2 \in \mathbb{R}^3$ *and angles* $\gamma_1, \gamma_2 \in \mathbb{R}$ *we have*

$$M(\gamma_1 \mathbf{a}_1)M(\gamma_2 \mathbf{a}_2) = M(\gamma_2 M(\gamma_1 \mathbf{a}_1)\mathbf{a}_2)M(\gamma_1 \mathbf{a}_1), \tag{S1}$$

where $M(\cdot)$ *is defined as equation (13).*

Proof. To prove it, we will use the fact that the cross product $\mathcal{K}(\mathbf{a})\mathbf{b}$, where $\mathcal{K}(\cdot)$ is defined in equation (14), is invariant under rotations $\mathbf{M}$

$$\mathbf{M}\mathcal{K}(\mathbf{a})\mathbf{b} = \mathcal{K}(\mathbf{M}\mathbf{a})\mathbf{M}\mathbf{b}, \tag{S2}$$

for all $\mathbf{a}, \mathbf{b} \in \mathbb{R}^3$ and any unitary matrix $\mathbf{M}$ defined as equation (13). This is true since the vector $\mathcal{K}(\mathbf{a})\mathbf{b}$ is orthogonal to $\mathbf{a}$ and $\mathbf{b}$ and its length depends on the area given by the parallelogram formed by $\mathbf{a}$ and $\mathbf{b}$. These properties are invariant under rotations, hence so is the cross product. Consequently,

$$\mathbf{M}\mathcal{K}(\mathbf{a}) = \mathcal{K}(\mathbf{M}\mathbf{a})\mathbf{M}. \tag{S3}$$

Now let us denote $\mathbf{v} = M(\gamma_1 \mathbf{a}_1)\mathbf{a}_2$ and, based on equation (S3), we can write

$$\begin{aligned}\mathcal{K}(\mathbf{v})M(\gamma_1 \mathbf{a}_1) &= \mathcal{K}(M(\gamma_1 \mathbf{a}_1)\mathbf{a}_2)M(\gamma_1 \mathbf{a}_1)\\ &= M(\gamma_1 \mathbf{a}_1)\mathcal{K}(\mathbf{a}_2). \end{aligned}\tag{S4}$$

By applying equation (S4) twice, we obtain

$$\begin{aligned}\mathcal{K}(\mathbf{v})^2 M(\gamma_1 \mathbf{a}_1) &= \mathcal{K}(\mathbf{v})M(\gamma_1 \mathbf{a}_1)\mathcal{K}(\mathbf{a}_2)\\ &= M(\gamma_1 \mathbf{a}_1)\mathcal{K}(\mathbf{a}_2)^2. \end{aligned}\tag{S5}$$

The right-hand side of equation (S1) can be simplified as

$$\begin{aligned}M(\gamma_2 M(\gamma_1 \mathbf{a}_1)\mathbf{a}_2)M(\gamma_1 \mathbf{a}_1) &= M(\gamma_2 \mathbf{v})M(\gamma_1 \mathbf{a}_1)\\ &= (\mathbf{I}_3 + \sin(2\gamma_2)\mathcal{K}(\mathbf{v}) + (1-\cos(2\gamma_2))\mathcal{K}(\mathbf{v})^2)M(\gamma_1 \mathbf{a}_1) \quad \text{(S6a)}\\ &= M(\gamma_1 \mathbf{a}_1) + \sin(2\gamma_2)\mathcal{K}(\mathbf{v})M(\gamma_1 \mathbf{a}_1) + (1-\cos(2\gamma_2))\mathcal{K}(\mathbf{v})^2 M(\gamma_1 \mathbf{a}_1)\\ &= M(\gamma_1 \mathbf{a}_1) + \sin(2\gamma_2)M(\gamma_1 \mathbf{a}_1)\mathcal{K}(\mathbf{a}_2) + (1-\cos(2\gamma_2))M(\gamma_1 \mathbf{a}_1)\mathcal{K}(\mathbf{a}_2)^2 \quad \text{(S6b)}\\ &= M(\gamma_1 \mathbf{a}_1)(\mathbf{I}_3 + \sin(2\gamma_2)\mathcal{K}(\mathbf{a}_2) + (1-\cos(2\gamma_2))\mathcal{K}(\mathbf{a}_2)^2)\\ &= M(\gamma_1 \mathbf{a}_1)M(\gamma_2 \mathbf{a}_2), \quad \text{(S6c)}\end{aligned}$$

where equations (S6a) and (S6c) follow from equation (13) and in equation (S6b) we used equations (S4) and (S5). □

Now we have the necessary tools to prove Theorem 1 in the main paper.

Proof. The theorem can be proved by showing that $M(\beta\mathbf{x})\mathbf{y} \sim \mathbf{y}$ for any real angle β. Let $\mathbf{z} = M(\beta\mathbf{x})\mathbf{y}$, which can be expressed as

$$\begin{aligned}\mathbf{z} &= M(\beta\mathbf{x})M(\gamma\mathbf{a})\mathbf{x} \\ &= M(\gamma M(\beta\mathbf{x})\mathbf{a})M(\beta\mathbf{x})\mathbf{x} \qquad \text{(S7a)} \\ &= M(\gamma M(\beta\mathbf{x})\mathbf{a})\mathbf{x}, \qquad \text{(S7b)}\end{aligned}$$

where in equation (S7a) we used Lemma 1. Since the vector $\mathbf{a}$ is uniformly distributed over the 3D sphere, the vector $M(\beta\mathbf{x})\mathbf{a}$ is also uniformly distributed over the 3D sphere [1, Def. 6.18], which makes $\mathbf{z} \sim \mathbf{y}$. □

II 3D Distribution Analysis

In this section, we derive the approximate pdf of the point $\mathbf{s_r} = M(\dot{\boldsymbol{\alpha}})\mathbf{s_u}$ for a fixed $\mathbf{s_u}$ and random $M(\dot{\boldsymbol{\alpha}})$. Exact expressions are very difficult to obtain, therefore we make use of the approximations $\sin 2\theta \approx 2\theta$ and $\cos 2\theta \approx 1$, valid for $\sigma_p^2 \ll 1$, i.e., $\|\dot{\boldsymbol{\alpha}}\| \ll 1$. Thus $M(\boldsymbol{\alpha})$ in equation (13) can be approximated as

$$\begin{aligned}M(\boldsymbol{\alpha}) &\approx \mathbf{I}_3 + 2\theta\mathcal{K}(\mathbf{a}) \\ &\approx \begin{pmatrix} 1 & -2\theta a_3 & 2\theta a_2 \\ 2\theta a_3 & 1 & -2\theta a_1 \\ -2\theta a_2 & 2\theta a_1 & 1 \end{pmatrix} \\ &\approx \begin{pmatrix} 1 & -2\alpha_3 & 2\alpha_2 \\ 2\alpha_3 & 1 & -2\alpha_1 \\ -2\alpha_2 & 2\alpha_1 & 1 \end{pmatrix}. \qquad \text{(S8)}\end{aligned}$$

Without loss of generality, we simplify the analysis by setting $\mathbf{s_u} = (1,0,0)^{\mathrm{T}}$. In this case, based on equation (S8), $\mathbf{s_r} = M(\dot{\boldsymbol{\alpha}})\mathbf{s_u} = (1, 2\dot{\alpha}_3, -2\dot{\alpha}_2)^{\mathrm{T}}$ and it can be noted that $\mathbf{s_r}$ then has a bivariate Gaussian distribution on the plane normal to $\mathbf{s_u}$ and the peak of the distribution centred at $\mathbf{s_u}$.

Using equation (S8) and by removing high order terms, such as $\alpha_i\alpha_j$ for any i, j, the multiplication of two matrices $M(\boldsymbol{\alpha})$ can be approximated as

$$\begin{aligned}M(\boldsymbol{\alpha})M(\boldsymbol{\beta}) &\approx \begin{pmatrix} 1 & -2\alpha_3 - 2\beta_3 & 2\alpha_2 + 2\beta_2 \\ 2\alpha_3 + 2\beta_3 & 1 & -2\alpha_1 - 2\beta_1 \\ -2\alpha_2 - 2\beta_2 & 2\alpha_1 + 2\beta_1 & 1 \end{pmatrix} \\ &\approx M(\boldsymbol{\alpha} + \boldsymbol{\beta}). \qquad \text{(S9)}\end{aligned}$$

From equation (S9) we can conclude that two consecutive small innovations can be replaced by a single innovation, i.e., $M(\dot{\boldsymbol{\alpha}}_1)M(\dot{\boldsymbol{\alpha}}_2)\mathbf{s_u} \sim M(\dot{\boldsymbol{\alpha}}_\mathrm{t})\mathbf{s_u}$, by doubling the variance σ_p^2.

III Autocorrelation

In this section, we derive the ACF of the SOP drift in equation (20). The derivation uses the Jones description of the model but the result is valid for the 4D description as well.

At first we will calculate the expectation of the innovation matrix from equation (2)

$$\begin{aligned}\mathbb{E}[J(\dot{\boldsymbol{\alpha}})] &= \mathbb{E}[\mathbf{I}_2\cos(\theta) - i(a_1\boldsymbol{\sigma}_1 + a_2\boldsymbol{\sigma}_2 + a_3\boldsymbol{\sigma}_3)\sin(\theta)] \\ &= \mathbb{E}[\cos(\theta)]\mathbf{I}_2 \qquad \text{(S10a)} \\ &= \int_0^\infty \cos(\theta) f_\theta(\theta)\mathrm{d}\theta\mathbf{I}_2 \\ &= \Big((1-\sigma_p^2)\exp\Big(-\frac{\sigma_p^2}{2}\Big)\Big)\mathbf{I}_2, \qquad \text{(S10b)}\end{aligned}$$

where $\dot{\boldsymbol{\alpha}} = (\dot{\alpha}_1, \dot{\alpha}_2, \dot{\alpha}_3) \sim \mathcal{N}(\mathbf{0}, \sigma_p^2\mathbf{I}_3)$, $\theta = \|\dot{\boldsymbol{\alpha}}\|$ and $\mathbf{a} = \dot{\boldsymbol{\alpha}}/\theta = (a_1, a_2, a_3)$. Equation (S10a) follows because $\mathbb{E}[a_i] = 0$ and the random variables a_i, θ are independent. Equation (S10b) follows because the probability density function (pdf) of θ is [2, eq. (3.195)]

$$f_\theta(\theta) = \frac{1}{\sigma_p^3}\sqrt{\frac{2}{\pi}}\theta^2\exp\Big(-\frac{\theta^2}{2\sigma_p^2}\Big), \qquad \text{(S11)}$$

for $\theta \geq 0$.

The ACF of $\mathbf{r}_k$ at time separation $l \geq 0$ for a constant input $\mathbf{u}$ is

$$\begin{aligned}\mathcal{A}_\mathbf{r}(k, k+l) &= \mathbb{E}[\mathbf{r}_k^\mathrm{H}\mathbf{r}_{k+l}] \\ &= \mathbf{u}^\mathrm{H}\mathbb{E}[\mathbf{J}_k^\mathrm{H}\mathbf{J}_{k+l}]\mathbf{u} \\ &= \mathbf{u}^\mathrm{H}\mathbb{E}[\mathbf{J}_k^\mathrm{H}J(\dot{\boldsymbol{\alpha}}_{k+l})\ldots J(\dot{\boldsymbol{\alpha}}_{k+1})\mathbf{J}_k]\mathbf{u} \\ &= \mathbf{u}^\mathrm{H}\mathbb{E}[J(\dot{\boldsymbol{\alpha}})]^l\mathbb{E}[\mathbf{J}_k^\mathrm{H}\mathbf{J}_k]\mathbf{u} \qquad \text{(S12a)} \\ &= \mathbf{u}^\mathrm{H}\mathbb{E}[J(\dot{\boldsymbol{\alpha}})]^l\mathbf{u}. \qquad \text{(S12b)}\end{aligned}$$

In equation (S12a) we used the fact that the expectation of the innovation matrix is a scaled identity matrix (equation (S10b)) that commutes with $\mathbf{J}_k^\mathrm{H}$, and the fact that the innovation matrices $J(\dot{\boldsymbol{\alpha}}_k)$ are independent.

Using equation (S10b) in equation (S12b), the ACF can be expressed as

$$\begin{aligned}\mathcal{A}_\mathbf{r}(l) &= \mathbf{u}^\mathrm{H}\Big(\Big((1-\sigma_p^2)\exp\Big(-\frac{\sigma_p^2}{2}\Big)\Big)\mathbf{I}_2\Big)^l\mathbf{u} \\ &= \|\mathbf{u}\|^2\Big((1-\sigma_p^2)\exp\Big(-\frac{\sigma_p^2}{2}\Big)\Big)^{|l|}. \qquad \text{(S13)}\end{aligned}$$

For symmetry reasons, the absolute value of $|l|$ replaced l in equation (S13), making the expression valid for negative l as well.

References

[1] A. Lapidoth and S. M. Moser, "Capacity bounds via duality with applications to multiple-antenna systems on flat-fading channels," *IEEE Transactions on Information Theory*, vol. 49, pp. 2426–2467, Oct. 2003.

[2] J. J. Shynk, *Probability, Random Variables, and Random Processes: Theory and Signal Processing Applications*. Hoboken, NJ: John Wiley & Sons, 2013.
